# Detection of Volatiles from Raw Beef Meat from Different Packaging Systems Using Solid-Phase Microextraction GC–Accurate Mass Spectrometry

**DOI:** 10.3390/foods10092018

**Published:** 2021-08-27

**Authors:** Debarati Bhadury, Yada Nolvachai, Philip J. Marriott, Joanne Tanner, Kellie L. Tuck

**Affiliations:** 1 School of Chemistry, Monash University, Wellington Rd, Clayton, VIC 3800, Australia; debarati.bhadury@monash.edu; 2 Bioresource Processing Research Institute of Australia (BioPRIA), Department of Chemical Engineering, Monash University, Alliance Ln, Clayton, VIC 3800, Australia; joanne.tanner@monash.edu; 3 Australian Centre for Research on Separation Science, School of Chemistry, Monash University, Wellington Rd, Clayton, VIC 3800, Australia; yada.nolvachai@monash.edu (Y.N.); philip.marriott@monash.edu (P.J.M.)

**Keywords:** GC–accQTOFMS, raw beef, headspace, SPME, meat quality

## Abstract

The volatile profile of raw beef contains vital information related to meat quality and freshness. This qualitative study examines the effect of packaging system on the formation and release of volatile organic compounds (VOCs) from raw beef over time, relative to the packaging best before date (BBD). The three packaging systems investigated were modified atmospheric packaging, vacuum packaging, and cling-wrapped packaging. Porterhouse steak samples with the same BBD were analysed from 3 days before to 3 days after the BBD. VOCs were detected via preconcentration using solid-phase microextraction combined with gas chromatography–accurate mass quadrupole time-of-flight mass spectrometry. In total, 35 different VOCs were tentatively identified. Interestingly, there was no clear relationship of the VOCs detected between the three packaging systems, with only carbon disulphide and acetoin, both known volatiles of beef, detected in all three. This is the first study to investigate the effects of commercial packaging systems on VOC formation; it provides an understanding of the relationship of VOCs to the BBD that is essential for the development of on-pack freshness and quality sensors.

## 1. Introduction

Due to its nutritional quality, meat is an important component of the daily diet in many countries. Meat belongs to the class of perishable food products, which are highly susceptible to spoilage and contamination due to various intrinsic and extrinsic factors, including microbial contamination, pH, temperature, and food packaging or handling [1,2]. Volatile organic compounds (VOCs), which are released as gases from the meat, are indicative of spoilage [2]. Commercially packaged meat products have a conservative advertised best before date (BBD) to ensure customers consume the product prior to spoilage. It should be noted that the BBD is an estimate, not an accurate indicator of freshness or quality.

The typical VOCs associated with raw meat and cooked samples include biogenic amines, sulphurous compounds, aldehydes, alcohols, nitrogenous compounds, ketones, hydrocarbons, and acids (Table S1). Solid-phase microextraction (SPME) combined with an appropriate gas chromatography technique has been used to detect VOCs [3,4]. SPME is inexpensive, relatively fast, easy-to-use, solvent free, and has the ability to simultaneously extract and pre-concentrate volatile compounds. Through a suitable interface design, it is compatible with a wide range of analytical instruments, though is mostly used for gas chromatography (GC) sample introduction [5]. Over 100 papers have investigated the analyte profile of cooked beef using SPME, or an alternate sampling technique. Recently, a small number of studies have employed headspace SPME with GC coupled to mass spectrometry (MS) to analyse volatile compounds from raw beef [4,6,7].

Whilst studies on cooked beef have provided an understanding of the effect that temperature, pH, and the addition of spices can have on the formation and release of VOCs, as well as the different pathways responsible for production of VOCs under various conditions [8], there are only a handful of reports that profile the analytes released from raw beef meat over storage time; the effect of packaging systems on the formation of volatile analytes over time is missing from the literature.

**Figure 1:**
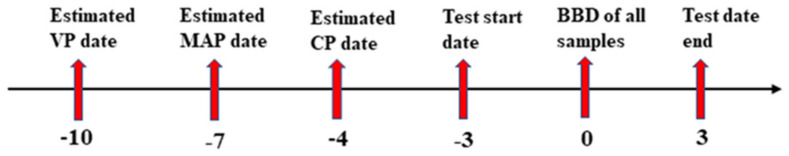
The estimated date of packaging of the beef steak samples and sampling time points for each of the packaging systems.

The packaging systems investigated in this work were modified atmosphere packaging (MAP), vacuum packaging (VP), and cling-wrapped packaging (CP). In MAP, which is a rigid package with a flexible film seal containing an atmosphere of 80% O_2_ and 20% CO_2_, a shelf life of 6–10 days after packaging can be achieved [9]. In CP, the meat is packaged in store using a low-density polyethylene thin film and the packaged product has a shorter shelf life of only 2–3 days. VP, in which the meat is placed in a vacuum environment, leads to a prolonged shelf life of (typically) 10–12 days, although up to 10 weeks is possible [10] (Figure 1). Regardless of the packaging type, the BBD noted on the pack is typically a conservative date, to ensure that the meat is eaten when freshness, quality, and taste are assured. The aim of this study was to investigate the detection of VOCs in different packaging formats, by using SPME and GC coupled to an accurate mass quadrupole time-of-flight MS (GC–accQTOFMS). The meat samples were analysed without any heat treatment and stored at 4 °C prior to analysis so that information about the compounds released by raw meat stored under refrigerated conditions could be obtained. The information about the VOC could be used to develop a freshness sensor for raw meat stored under refrigerated conditions.

## 2. Materials and Methods

### 2.1. Meat Samples

Porterhouse steaks were chosen as the standard cut of beef for this investigation due to their wide popularity and commercial availability, and the variety of packaging systems in which they are sold in the Australian supermarket. Three packaged raw porterhouse beef steaks, also known as sirloin steaks or New York steaks, were purchased from the same supermarket with identical BBD. The steaks were packed in three types of packaging systems, (1) MAP (containing 80% O_2_ and 20% CO_2_), (2) VP, and (3) CP. Four days prior to the printed BBD, each beef steak was diced and 10 ± 0.02 g aliquots were placed in 20 mL vials which were purged with nitrogen gas for 20 s and immediately sealed to prevent the saturation of oxygen in the headspace [11]. All vials were stored at 4 °C until analysis. For each packaging system, triplicate samples were prepared for daily analysis over a 7-day period (from 3 days before to 3 days after the BBD, *n* = 21 subsamples for each packaging system). The origin of the meat and packaging parameters could not be obtained due to the complex supply chain process and lack of information regarding chain of custody. The focus of the study is on the identification and qualitative analysis of volatiles released from meat stored in different packaging systems, rather than on the quantification of the individual VOCs relative to the provenance and history of the meat itself. Therefore, this research can be described as a consumer awareness study regarding packaging systems, and information on meat origin was deemed not to be required

### 2.2. SPME Method

SPME sampling was conducted using a triple-phase divinylbenzene/carboxen/polydimethylsiloxane (DVB/CAR/PDMS, 30 µm, grey) fibre (Supelco, Bellefonte, PA, USA). The fibre was conditioned by heating in a SPME Fibre Conditioning Station at 250 °C for 30 min. The sample was removed from the refrigerator, and SPME begun immediately. The fibre was introduced into the sample headspace and volatile analytes were preconcentrated on the fibre, without stirring, for a total extraction time of 20 min.

### 2.3. Method Development

GC coupled to accQTOFMS was used for analysis, allowing the determination of accurate mass (up to 4 decimal places). Analytes were identified by NIST library database matching, retention index (*I* < 20), and mass accuracy (M.A. < 20 ppm). During method development, various SPME and headspace injection methods were performed. Due to improved signal-to-noise (S/N) ratio, the SPME technique combined with GC–accQTOFMS analysis was chosen for all future analyte profiling. As for fibre selection, 30 µm DVB/CAR/PDMS fibre was chosen based on the literature, where it was used for the determination of the aroma profile of cooked beef [12]. After each injection, the SPME fibre was cleaned in an SPME conditioning station at 250 °C for 15 min to ensure that the fibre was completely clean before the next extraction. A blank SPME injection (SPME fibre without sample) was performed before every sample (see Figure S1 for a representative GC–MS trace). The peak at around 21.5 min is due to SPME fibre bleed. SPME sampling, 20 min, commenced immediately on removal of the samples from the refrigerator (4 °C).

### 2.4. GC–MS Analysis

Volatile compounds extracted using the SPME method were analysed by an Agilent gas chromatograph (7890A) equipped with a HP-5MS capillary column (30 m length × 0.25 mm I.D. × 0.25 µm *d*_f_) and a 7200 accQTOFMS (Agilent Technologies, Mulgrave, VIC, Australia). Injection port temperature is 175 °C with splitless injection (split vent flow 50 mL/min from 0.5 min). The SPME fibre desorption time was 5 min. Helium (UHP, 99.999%) was used as a carrier gas with a constant flow rate of 1 mL/min. The oven temperature was held at 33 °C for 5 min, then ramped at 10 °C/min to a final temperature of 200 °C, where it was held for 5 min. The transfer line temperature was 200 °C. MS quadrupole and ion source temperatures were 150 and 230 °C, respectively. Total analysis time was 26 min. The MS was operated in full scan mode with a scanning range of *m*/*z* 30–400. The data acquisition rate was 50 spectra/s. Since this system is normally used for comprehensive two-dimensional gas chromatography, which generates very narrow peak widths, 50 Hz is our default setting, and was not adjusted for this specific study. Whilst this will decrease mass accuracy, the ion mass can still be reliably used to predict ion empirical formulae for compounds reported here. Data processing was performed using MassHunter Qualitative Analysis 10.0 (Agilent Technologies, Mulgrave, VIC, Australia). Compound identification was achieved using the NIST10 mass spectrum library, retention index (*I*), and mass accuracy. *I* (retention index) was calculated based on the elution times of *n*-alkane standards C7–C20 analysed under identical GC–MS parameters for the calculation of the Kovatś Index values of the compounds.

## 3. Results and Discussion

### 3.1. VOC Profile and Identification

Guided by previous reports of the use of SPME for concentration of volatiles, two fibres, DVB/CAR/PDMS and DVB/PDMS, were investigated for method development [13,14]. Based on the results of the experiment, DVB/CAR/PDMS fibres with 20 min extraction time at room temperature were chosen as best suited for the study. Further studies with DVB/CAR/PDMS fibres showed that a 20 min sampling period resulted in good signal-to-noise (S/N) ratios and after this time the sample temperature was 10 °C. Shorter sampling times resulted in poor S/N ratios, whilst longer sampling times allowed the sample temperature to rise above 10 °C, which was deemed unacceptable as it could result in an analyte profile that was not consistent with the sample being stored in a refrigerated environment. Previous studies have shown that elevated equilibration temperatures and longer SPME sorption time (e.g., 37 °C for 60 min [15] and 80 °C for greater than 50 min [4]) result in the formation of artefact analytes. Hence, one of the key findings in this study is that cold extraction was sufficient for detailed analyte profile determination, at the same time allowing a direct correlation to be made with the analyte profile of meat samples stored at 4 °C. This study focused on identifying volatile compounds as they are more relevant to the freshness of meat, whereas non-volatile compounds are typically related to the flavour profile.

Tentative identification of VOCs was achieved following SPME-GC–accQTOFMS analysis by a combination of several matching methods. Compounds were initially screened by matching the mass spectrum of each peak in the experimental GC trace with the list of compound mass spectra from the NIST library. The match was accepted if the differences between the experimental and literature retention index and mass accuracy were within ±20 and ±20 ppm, respectively. The 35 compounds tentatively identified by this method are presented in Table 1. Example GC–MS traces are shown in Figures S2–S4 and the compounds corresponding to the respective packaging systems are included in tables below the chromatograms (Tables S2–S4). The compounds of raw beef that have been previously reported are summarised in Table S1. Of the 35 compounds reported in this study (Table 1), 29 were detected with a mass accuracy of below 10 ppm with 27 previously identified in the literature as VOCs from either cooked or raw meat. For seven of the compounds in Table 1, only the mass accuracy criterion was able to be met for tentative identification. Of these, carbon disulphide (CS_2_) [6,7,16], acetic acid [6,17,18], ethyl acetate [19,20], acetaldehyde [6,21], and hexanal [6,7,22] have been previously identified in chemical analysis of beef and the tentative match was therefore accepted. Six compounds from this study were able to be identified only by molecular features (hydrocarbon or alcohol-containing) and five of the peaks observed in the GC experimental results were unable to be identified (Table S2). The literature-supported identification of volatile compounds released from raw beef (Table 1), as well as information about the structural features and chemical functionality of some of the compounds which were unable to be further identified (Table S5), combined with the packaging system from which these samples originated, can be useful for future applications, including freshness sensor design.

Previous studies attributed many of the VOCs detected in the analysis of cooked meat to heat-induced Maillard reactions, or to the oxidation of lipids at the elevated temperature [47]. However, as we observed these VOCs from raw beef without any heat treatment, it can be concluded that heat is not the only factor responsible for the presence of these compounds. This is in agreement with the findings of Insausti et al. [21], where dimethyl sulphide was shown to be a degradation product formed during storage of meat at low temperature. Interestingly, whilst many of the analytes reported by Bueno et al. [3] were observed, they were typically present at <1% of the relative response area in the current study (with the exception of hexanal and 2,3-octanedione) and thus have not been included in Table 1. Bueno et al. preconcentrated their samples on a PDMS/DVD fibre at 37 °C for 40 min, thus resulting in the detection of a greater quantity of these VOC as well as possible degradation products which were undetected or present only at low concentrations in the present study.

### 3.2. Comparison of Analyte Profiles from Different Packaging Systems

**Figure 2:**
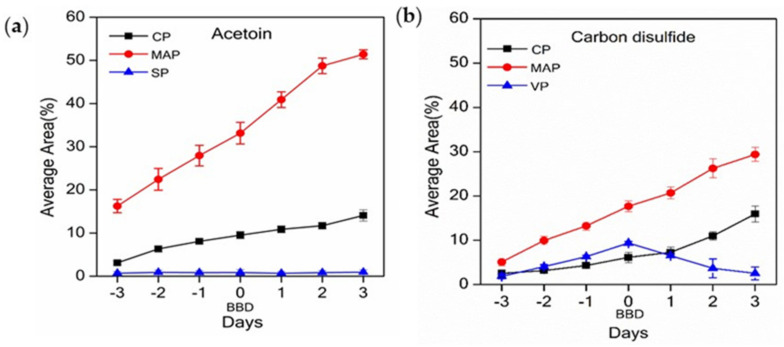
The average area, expressed as a percentage of all analytes, of (**a**) acetoin and (**b**) carbon disulphide (CS_2_); cling-wrapped packaging (CP), modified atmospheric packaging (MAP), and vacuum packaging (VP); *n* = 3 ± S.E.

Three VOCs were common to all packaging systems; CS_2_, acetoin, (Figure 2a,b), and 2-vinyloxyethanol (presumably an impurity from the packaging system) (Figure S5). Four common VOCs were observed in two out of three packaging systems: 2,3-butanediol was detected in samples from MAP and CP systems (Figure S6), 7-ethyl-1,3,5-cycloheptatriene (Figure S7), 1,3-bis(1,1-dimethyl-ethyl)benzene (Figure S8), and hexanal (Figure S9) were detected in samples from MAP and VP systems; and toluene was detected in samples from CP and VP systems (Figure S10).

Whilst the method employed in this study does not allow quantitative analysis as conducted, comparisons between packaging systems over the sampling period can be made by comparing the area of the relevant ion peak (taken as the average of the area each triplicate daily sample), expressed as a percentage of all analytes observed for that analysis. Higher relative abundance of CS_2_ and acetoin detected in beef samples from MAP and CP were observed with increased storage time. In contrast, samples from VP showed an initial increase in the amount of CS_2_ and acetoin, before reaching a maximum on the BBD and then decreasing with continued storage (Figure 2). Variations in the daily rates of change are attributed to the differences in butchery dates and packaging systems. As the true age of the samples differs, it is not unexpected that the stage of degradation and concentration of analytes also differs, and, hence, the difference in number and identity of analytes detected in beef samples from different packaging systems.

The aroma of raw beef during refrigerated storage is primarily due to the presence of CS_2_ [46]. Hence, as expected, CS_2_ was detected in all three packaging systems. However, this compound has been identified in only three of the previous studies [6,7,16], which could be due to that fact that there are few reports that investigate the volatiles from raw beef. Sulphurous compounds are formed due to the breakdown of the amino acids cysteine and methionine, which in turn are produced from proteins present in meat by microbes that are likely introduced at the slaughterhouse [48].

The detection of acetoin in all three packaging formats was also expected. Acetoin is reported to be a VOC of raw meat and can form via a number of pathways; decarboxylation of α-acetolactate, synthesis from aspartate in the presence of α–ketoglutarate [49], or production via a multistep enzymatic pathway in which *Leuconoctoc gasicomitatum* initially converts aspartate to oxaloacetate, which is then converted to acetoin via oxaloacetate decarboxylase [50]. Thus, acetoin can reasonably be assumed as a microbial by-product. As acetoin can be converted to 2,3-butanediol in the presence of 2,3-butanediol dehydrogenase [50], the presence of 2,3-butanediol was also expected and was observed in both MAP and CP samples (Table 1).

2-Vinyloxyethanol was also detected in all three packaging systems. This is not a known volatile released from raw beef during storage and hence is assumed to be a contamination from meat packaging, which contains polymerised forms of vinyl alcohol [51]. Ethylene oxide, on the other hand, observed only in meat from MAP packaging, is assumed to have been formed from endogenous ethylene as a result of microbial growth. A high number of hydrocarbons was observed; Table 1 notes if they have been previously reported. Hydrocarbons are associated with oxidative pathways in meat and thus the number observed is not unexpected.

The relative abundance of the volatiles CS_2_ and acetoin (see Figure 2) and 2-vinyloxyethanol (see Figure S5) varied significantly at certain timepoints among the three different packaging systems. The difference in the analyte abundance between the packaging systems is likely due to the different internal environments and duration of meat storage inside the package prior to sampling and analysis. Other factors affecting reaction rate may include endogenous microbial activity [52], and chemical reactions between the components present in the food matrix. These compounds are therefore indicative of the extent of spoilage of the raw beef in each packaging system, and their presence, absence, and concentration could be used as an indication of raw meat quality and freshness.

Overall, 14, 15, and 17 VOCs were detected in raw beef meat in MAP, CP, and VP, respectively, over the 7-day sampling period. Many compounds were unique to a single packaging system. It is clear that the packaging environment plays a significant role in the volatile profile of the packaged raw beef. Investigation of the biological pathways and organisms responsible for the production of volatile compounds from raw beef was outside the scope of this study and the exact reagents, biological entities, and sources of the majority of the analytes, as well as the differences in their presence, absence, or concentration in a different packaging environment, are still unknown. The knowledge from this qualitative, comparative study allows a better understanding of release profiles in different packaging systems, which is necessary in order to design an on-pack sensor for freshness detection.

## 4. Conclusions

The volatile profiles of raw beef steak samples initially packaged in MAP, VP, and CP were determined over a 7-day period. SPME combined with GC–accurate mass QTOFMS was utilised for sample analysis, and 35 volatile compounds were identified across the three packaging systems. Two compounds (acetoin and carbon disulphide) and one contaminant (2-vinyloxyethanol) were detected in meat from all three packaging systems and a further five compounds (2,3-butanediol, 7-ethyl-1,3,5-cycloheptatriene, 1,3-bis(1,1-dimethyl-ethyl)benzene, hexanal, and toluene) were present in at least two systems. The trends in relative abundance of these compounds over time differed between packaging systems and can also possibly be related to packing dates. Many compounds that were previously reported from cooked meat were assumed to be generated by thermal processes; for instance, the Maillard reaction or lipid oxidation. The current work shows that these compounds are also released from packaged raw beef without any heat treatment. Therefore, it can be concluded that heat is not the primary release factor, and that the packaging conditions play an important role in the release profile of volatile compounds from raw beef. Many of the common compounds detected in this qualitative study are indicative of meat spoilage and information about their presence, absence, and abundance over time could be used to indicate packaged meat freshness. The demonstrated relationships between packaging systems, volatile compounds, and storage times therefore have potential application in the development of packaged raw meat freshness and quality sensors.

## Figures and Tables

**Table 1:** VOCs of raw porterhouse steak samples from modified atmospheric packaging (MAP), cling-wrapped packaging (CP), and/or vacuum packaging (VP) identified using SPME-GC–accQTOFMS. Analytes are sorted by packaging systems and retention time.

Tentative Compound Identification	Retention Time (min)	Packaging System	Characteristic	M.A.	*I*	References
			*m*/*z*	(ppm)	(∆*I* ^b^)	
			(fragment) ^a^			
carbon disulphide	2.70	MAP	77.6790	1.54	N/A	[6,7,16]
		CP			(N/A)	
(CS_2_)		VP				
acetoin	6.043	MAP	88.0520	8	739	[6,7,16,23,24]
		CP			(11)	
(C_4_H_8_O_2_)		VP				
2-vinyloxyethanol ^c^	5.83	MAP	88.0492	−10.44	728	Present work
(C_4_H_8_O_2_)		CP			(4)	
		VP				
2,3-butanediol ^d^	5.75	MAP	60.0207	−1.99	724	[16,25]
(C_4_H_10_O_2_)		CP	(C_2_H_4_O_2_)		(2)	
7-ethyl-1,3,5-cycloheptatriene ^d^	10.09	MAP	91.0542	0.81	966	[26]
(C_9_H_12_)		VP	(C_7_H_7_)		(8)	
1,3-bis(1,1-dimethyl-ethyl)benzene	17.35	MAP	190.1714	1.06	1360	[27]
(C_14_H_22_)		VP			(3)	
methylbenzene	7.4401	CP	92.06182	2.73	802	[8,28]
(toluene)		VP			(9)	
(C_7_H_8_)						
hexanal	2.89	MAP	100.172	3.50	N/A	[6,7,29,30]
(C_6_H_12_O)		VP			(N/A)	
ethylene oxide	3.26	MAP	52.8103	8.76	N/A	Present work
(C_2_H_4_O)					(N/A)	
3,3,4-trimethylhexane ^d,^^e^	6.91	MAP	99.1161	7.33	777	[31]
(C_9_H_2O_)			(C_7_H_15_)		(10)	
3-hydroxybutanal	7.13	MAP	88.0515	0.05	787	[32]
(acetaldol) ^d^					(6)	
(C_4_H_8_O_2_)						
3,4,5-trimethyl-heptane ^d^	7.59	MAP	85.1011	0.3	818	Present work
(C_10_H_22_)			(C_6_H_13_)		(5)	
1-nonene ^d,e^	8.79	MAP	97.0998	3.45	916	[33]
(C_9_H_18_)			(C_7_H_13_)		(10)	
2,3-octanedione ^d,e^	12.80	MAP	99.0800	4.45	1087	[3,34]
(C_8_H_14_O_2_)			(C_6_H_11_O)		(1)	
2,2-dimethyl-heptane ^d^	7.85	MAP	113.1327	1.97	844	Present work
(C_9_H_20_)			(C_8_H_17_)		(13)	
2,6-dimethyldecane ^d,e^	12.61	CP	113.1314	9.52	1078	[17]
(C_12_H_26_)			(C_8_H_17_)		(9)	
5-methyl-4-undecene ^d^	14.70	CP	97.1002	10.06	1190	Present work
(C_12_H_24_)			(C_7_H_13_)		(9)	
2,2,8-trimethyl-decane ^d,e^	13.72	CP	111.1163	4.74	1136	[35]
(C_13_H_28_)			(C_8_H_15_)		(15)	
2-propyl-1-heptanol ^d,e^	14.84	CP	126.1382	16.66	1014	[36]
(C_10_H_22_O)			(C_9_H_18_)		(14)	
decanal ^d^	14.96	CP	99.0816	−11.69	1205	[35,36]
(C_10_H_20_O)			(C_6_H_11_O)		(14)	
4-ethyl-2,2,6,6-tetramethyl-heptane ^d,e^	12.68	CP	99.1158	10.36	1081	[20]
(C_13_H_28_)			(C_7_H_15_)		(1)	
3-methyldecane ^d,e^	12.25	CP	112.1242	4.03	1061	[37]
(C_11_H_24_)			(C_8_H_16_)		(10)	
2,5-dimethyldecane ^d^	12.52	CP	98.1098	8.14	1074	Present work
(C_12_H_26_)			(C_7_H_14_)		(12)	
ethyl acetate	3.69	CP	77.6720	−10.44	N/A	[19,20]
(C_4_H_8_O_2_)					(N/A)	
heptane	5.23	CP	100.1239	7.51	799	[23]
(C_7_H_16_)					(2)	
acetic acid	3.36	VP	66.3920	6.34	N/A	[6,17,18]
(C_2_H_4_O_2_)					(N/A)	
3-methylene-heptane ^d^	6.79	VP	112.1236	9.3	771	[38]
(C_8_H_16_)					(11)	
1,2,4-trimethyl-cyclopentane	7.19	VP	112.1251	−4	789	[39]
(C_8_H_16_)					(6)	
3,4-dimethyl-1-octene ^d, e^	8.35	VP	85.1009	7.43	897	Present work
(C_10_H_20_)			(C_6_H_13_)		(20)	
2-heptanal	8.83	VP	112.1271	0.3	917	[40]
(C_7_H_12_O)					(4)	
3-methylnonane ^d, e^	10.46	VP	127.1487	4.51	980	[41]
(C_10_H_22_)			(C_9_H_19_)		(9)	
hydroxyurea	2.71	VP	77.2447	1.19	N/A	[42]
(CH_4_N_2_O_2_)					(N/A)	
3,7-dimethylnonane ^d, e^	10.53	VP	127.1458	10.44	983	[43,44,45]
(C_11_H_24_)			(C_9_H_19_)		(3)	
acetaldehyde	3.22	VP	46.421	2.37	N/A	[6]
(C_2_H_4_O)					(N/A)	
dimethyl disulphide	7.09	VP	93.992	5.82	785	[6,7,46]
(C_2_H_6_S_2_)					(3)	

^a^ Shown in the bracket is the ion *m*/*z* value used for identification if the molecular ion was not detected. ^b^ ∆*I* = |Experimental *I*—NIST Reference *I*|. The same stationary phase reference I value was used, but equivalent conditions cannot be guaranteed. ^c^ Analyte likely to arise from packaging. ^d^ Analyte identified from their fragment ions and the formula of corresponding fragment ions are given. Analyte peaks < 1% relative response area in the respective packaging system have not been included in this table. ^e^ Analytes have been previously reported. The fragment *m*/*z* ion, *I* and ∆*I* values, and comparison to NIST library database are supportive of the isomers noted.

## Data Availability

The data presented in this study are available on request from the corresponding author.

## Supplementary Materials

The following are available online at https://www.mdpi.com/article/10.3390/foods10092018/s1. Table S1: Summary of the volatile compounds detected from or associated with raw and cooked meat samples. Figure S1: Total ion chromatogram of blank sample. Figure S2: Total ion chromatogram of VOCs of raw beef steaks from MAP; the compounds identified are summarised in Table S2. Table S2: Volatile compounds identified in this study from MAP packaged raw beef steaks. Figure S3: Total ion chromatogram of VOCs of raw beef steaks from CP; the compounds identified are summarised in Table S3. Table S3: Volatile compounds identified in this study from CP packaged raw beef steaks. Figure S4: Total ion chromatogram of VOCs of raw beef steaks from VP; the compounds identified are summarised in Table S4. Table S4: Volatile compounds identified in this study from VP packaged raw beef steaks. Figure S5: The average area, expressed as a percentage of all analytes, of 2-(vinyloxy)ethanol; *n* = 3, ±S.E. Figure S6: The average area, expressed as a percentage of all analytes, of 2,3-butanediol; *n* = 3, ±S.E. Figure S7: The average area, expressed as a percentage of all analytes, of 7-ethyl-1,3,5-cycloheptatriene; *n* = 3, ±S.E. Figure S8: The average area, expressed as a percentage of all analytes, of 1,3-bis(1,1-dimethyl-ethyl)benzene; *n* = 3, ±S.E. Figure S9: The average area, expressed as a percentage of all analytes, of hexanal; *n* = 3, ±S.E. Figure S10: The average area, expressed as a percentage of all analytes, of toluene; *n* = 3, ±S.E. Table S5: Volatile compounds from this study which are identified only by molecular features (containing hydrocarbon or alcohol) along with peaks observed in the GC experimental results which were unable to be identified.
