# Supplementary material for: Detection of Volatiles from Raw Beef Meat from Different Packaging Systems Using Solid-Phase Microextraction GC–Accurate Mass Spectrometry
Source: Foods. 2021 Aug 27;10(9):2018. doi: 10.3390/foods10092018 (PMC8468586; doi:10.3390/foods10092018)
Supplement: Supplementary file 1 [file foods-10-02018-s001.zip › foods-1336114-supplementary.pdf]

SUPPORTING INFORMATION

DETECTION OF VOLATILES FROM RAW BEEF MEAT USING SOLID PHASE MICROEXTRACTION GC–ACCURATE MASS SPECTROMETRY METHOD.

Debarati Bhadury,[a,b] Yada Nolvachai,[c] Philip J. Marriott,[c,*] Joanne Tanner,[b,*] Kellie L. Tuck[a,*]

[a] School of Chemistry, Monash University, Clayton, VIC 3800, Australia.

[b] Bioresource Processing Research Institute of Australia (BioPRIA), Department of Chemical Engineering, Monash University, Clayton, VIC 3800, Australia.

[c] Australian Centre for Research on Separation Science, School of Chemistry, Monash University, Clayton, VIC 3800, Australia.

*Corresponding author email:kellie.tuck@monash.edu; Joanne.tanner@monash.edu; philip.marriott@monash.edu

Table S1. Summary of the volatile compounds detected from or associated with raw meat samples.

Figure S1. Total ion chromatogram of a SPME fibre bleed.

Figure S2. Total ion chromatogram of VOCs of raw beef steaks from MAP, the compounds identified are summarised in Table S2.

Table S2. Volatile compounds identified in this study from MAP packaged raw beef steaks.

Figure S3. Total ion chromatogram of VOCs of raw beef steaks from CP, the compounds identified are summarised in Table S3.

Table S3: Volatile compounds identified in this study from CP packaged raw beef steaks.

Figure S4. Total ion chromatogram of VOCs of raw beef steaks from VP, the compounds identified are summarised in Table S4.

Table S4: Volatile compounds identified in this study from VP packaged raw beef steaks.

Figure S5. The average area, expressed as a percentage of all analytes, of 2-(vinyloxy)ethanol; $n = 3$, ±S. E.

Figure S6. The average area, expressed as a percentage of all analytes, of 2,3-butanediol; $n = 3$, ±S. E.

Figure S7. The average area, expressed as a percentage of all analytes, of 7-ethyl-1,3,5-cycloheptatriene; $n = 3$, ±S. E.

Figure S8. The average area, expressed as a percentage of all analytes, of 1,3-bis(1,1-dimethyl-ethyl)benzene; $n = 3$, ±S. E.

Figure S9. The average area, expressed as a percentage of all analytes, of hexanal; $n = 3$, ±S. E.

Figure S10. The average area, expressed as a percentage of all analytes, of toluene; $n = 3$, ±S. E.

Table S5. Volatile compounds from this study which are identified only by molecular features (hydrocarbon or alcohol-containing) along with peaks observed in the GC experimental results which were unable to be identified.

Table S1. Summary of the volatile compounds detected from or associated with raw meat samples.

Compound class	Compounds Detected	References
Biogenic amines	Cadaverine	[1,2,3,4]
	Putrescine	[3,4]
	Tyramine	[1,4]
	Histamine	[3,4]
Sulfurous compounds	Carbon disulfide	[5]
	Dimethyl disulfide	[5]
	Hydrogen sulfide	[6]
	Methanethiol	[6,7]
Aldehydes	Hexanal	[8]
	Heptanal	[8]
	Acetaldehyde	[6,9]
	Benzaldehyde	[10]
	Nonanal	[8]
	Decanal	[11]
Alcohols	Ethanol	[10]
Nitrogenous compounds	2-Acetylthiazole	[12]
	Pyrazine	[13]
Ketones	2-Propanone	[14]
	2-Heptanone	[5,6,15]
Hydrocarbons	2,3,3-Trimethylpentane	[14]
	2,2,5-Trimethylhexane	[14]
	2-Octene	[14]
	3-Octene	[14]
	Nonene	[14]
	Tridecane	[16]
	3-Methyl-2-heptene	[9]
	Toluene	[17]
Acids	Acetic acid	[8]
	Butanoic acid	[10]
	Hexanoic acid	[10]

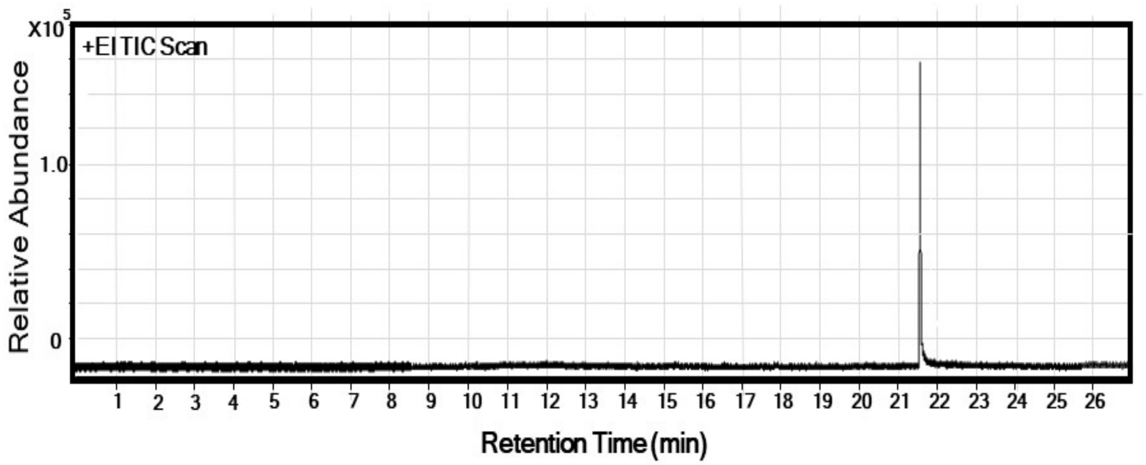

Figure S1. Total ion chromatogram of SPME fibre bleed.

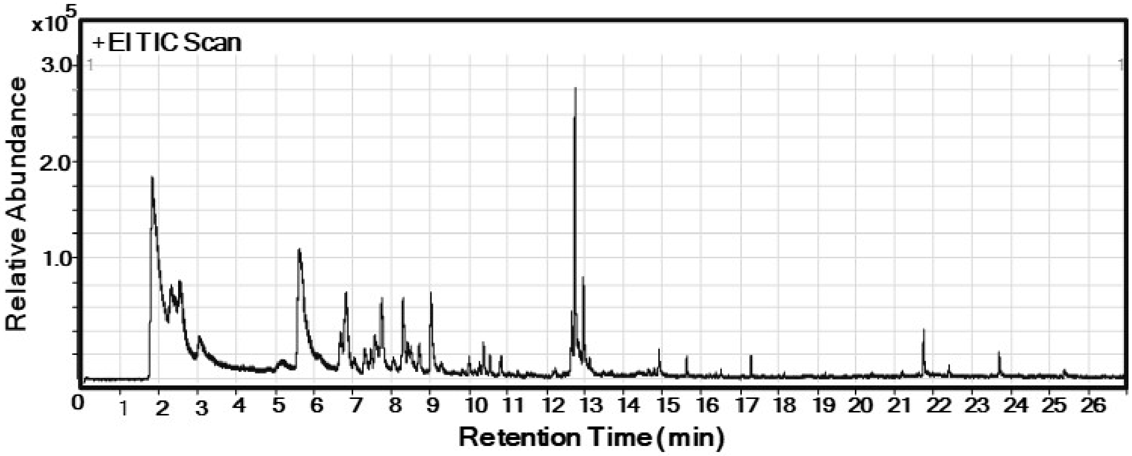

Figure S2. Total ion chromatogram of VOCs of raw beef steaks from MAP, the compounds identified are summarised in Table S2.

Table S2. Volatile compounds identified in this study from MAP packaged raw beef steaks.

Compounds	Retention Time
carbon disulfide (CS_2)	2.70
hexanal ($C_6H_{12}O$)	2.89
ethylene oxide (C_2H_4O)	3.26
2,3-butanediol ($C_4H_{10}O_2$)	5.75
2-vinyloxyethanol ($C_4H_8O_2$)	5.83
acetoin ($C_4H_8O_2$)	6.043
3,3,4-trimethylhexane (C_9H_{2O})	6.91
3-hydroxybutanal (acetaldol) ($C_4H_8O_2$)	7.13
3,4,5-trimethyl-heptane ($C_{10}H_{22}$)	7.59
2,2-dimethyl-heptane (C_9H_{20})	7.85
1-nonene (C_9H_{18})	8.79
7-ethyl-1,3,5-cycloheptatriene (C_9H_{12})	10.09
2,3-octanedione ($C_8H_{14}O_2$)	12.80
1,3-bis(1,1-dimethyl-ethyl)benzene ($C_{14}H_{22}$)	17.35

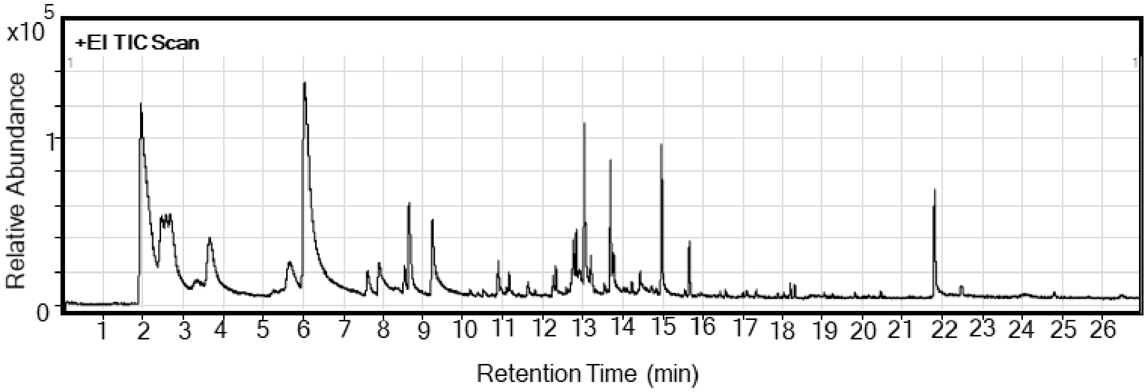

Figure S3. Total ion chromatogram of VOCs of raw beef steaks from CP, the compounds identified are summarised in Table S3.

Table S3: Volatile compounds identified in this study from CP packaged raw beef steaks.

Compounds	Retention Time
carbon disulfide (CS_2)	2.70
ethyl acetate ($C_4H_8O_2$)	3.69
heptane (C_7H_{16})	5.23
2,3-butanediol ($C_4H_{10}O_2$)	5.75
2-vinyloxyethanol ($C_4H_8O_2$)	5.83
acetoin ($C_4H_8O_2$)	6.043
methylbenzene (toluene) (C_7H_8)	7.4401
3-methyldecane ($C_{11}H_{24}$)	12.25
2,5-dimethyldecane ($C_{12}H_{26}$)	12.52
2,6-dimethyldecane ($C_{12}H_{26}$)	12.61
4-ethyl-2,2,6,6-tetramethyl-heptane ($C_{13}H_{28}$)	12.68
2,2,8-trimethyl-decane ($C_{13}H_{28}$)	13.72
4-methyl-5-undecene ($C_{12}H_{24}$)	14.70
2-propyl-1-heptanol ($C_{10}H_{22}O$)	14.84
decanal ($C_{10}H_{20}O$)	14.96

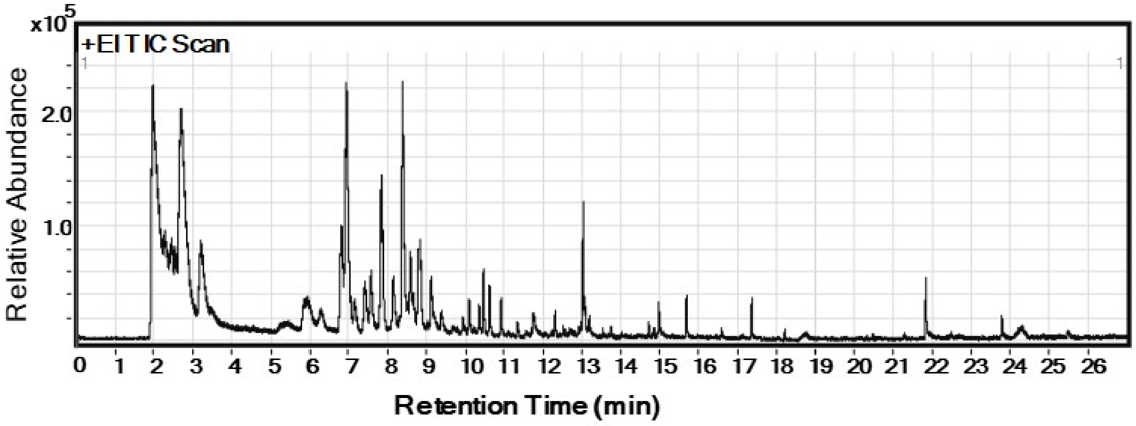

Figure S4. Total ion chromatogram of VOCs of raw beef steaks from VP, the compounds identified are summarised in Table S4.

Table S4: Volatile compounds identified in this study from VP packaged raw beef steaks.

Compounds	Retention Time
carbon disulfide (CS_2)	2.70
hexanal ($C_6H_{12}O$)	2.89
hydroxyurea ($CH_4N_2O_2$)	3.01
acetaldehyde (C_2H_4O)	3.22
acetic acid ($C_2H_4O_2$)	3.36
2-vinyloxyethanol[c] ($C_4H_8O_2$)	5.83
acetoin ($C_4H_8O_2$)	6.043
3-methylene-heptane[d] (C_8H_{16})	6.79
3,4-dimethylheptane[d] (C_9H_{20})	6.88
dimethyl disulfide ($C_2H_6S_2$)	7.09
1,2,4-trimethyl-cyclopentane (C_8H_{16})	7.19
methylbenzene (toluene) (C_7H_8)	7.4401
3,4-dimethyl-1-octene ($C_{10}H_{20}$)	8.35
2-heptanal[d] ($C_7H_{12}O$)	8.83
7-ethyl-1,3,5-cycloheptatriene (C_9H_{12})	10.09
3-methylnonane[d] ($C_{10}H_{22}$)	10.46
3,7-dimethylnonane[d] ($C_{11}H_{24}$)	10.53
1,3-bis(1,1-dimethyl-ethyl)benzene ($C_{14}H_{22}$)	17.35

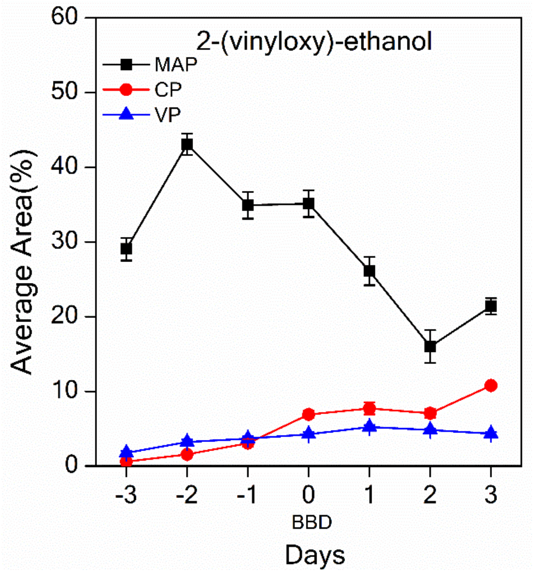

Figure S5. The average area, expressed as a percentage of all analytes, of 2-(vinyloxy)ethanol; $n = 3$, ±S. E.

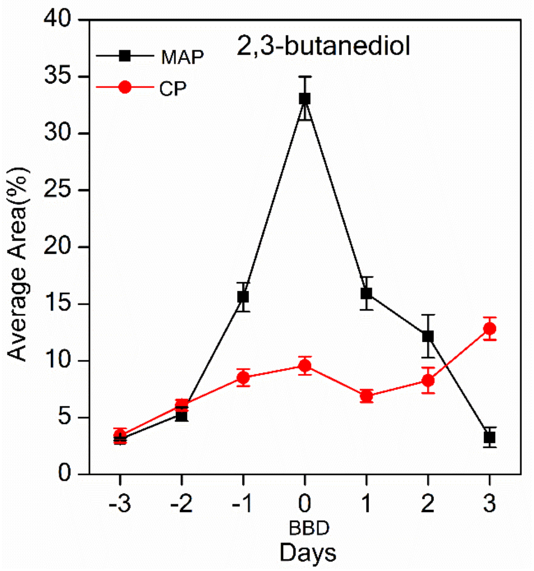

Figure S6. The average area, expressed as a percentage of all analytes, of 2,3-butanediol; $n = 3$, ±S. E.

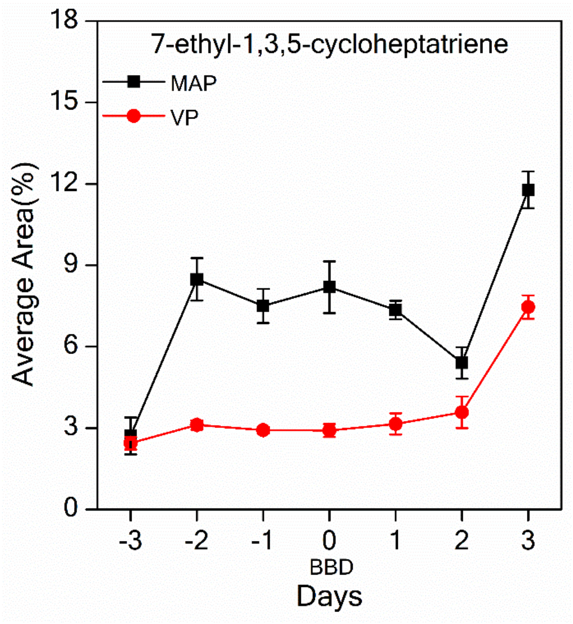

Figure S7. The average area, expressed as a percentage of all analytes, of 7-ethyl-1,3,5-cycloheptatriene; $n = 3$, ±S. E.

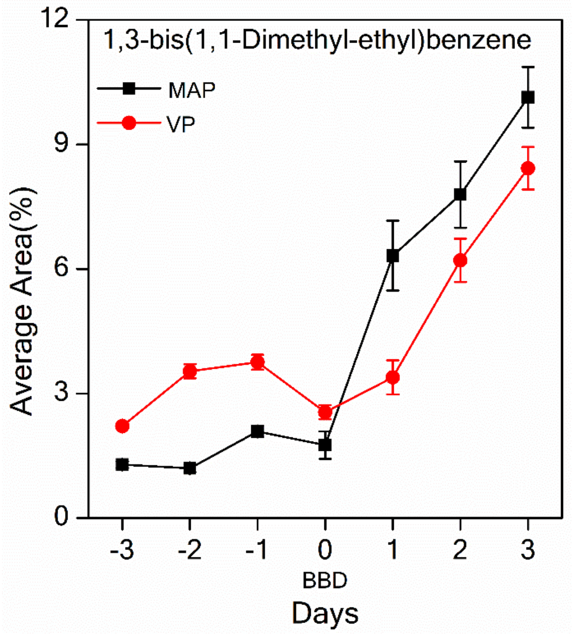

Figure S8. The average area, expressed as a percentage of all analytes, of 1,3-bis(1,1-dimethyl-ethyl)benzene; $n = 3$, ±S. E.

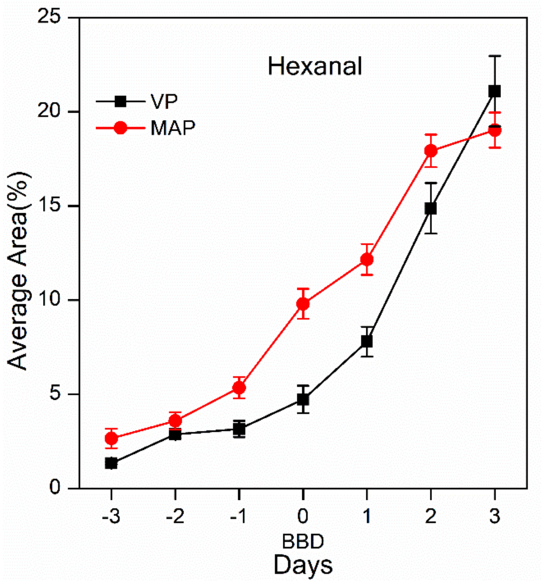

Figure S9. The average area, expressed as a percentage of all analytes, of hexanal; $n = 3$, ±S. E.

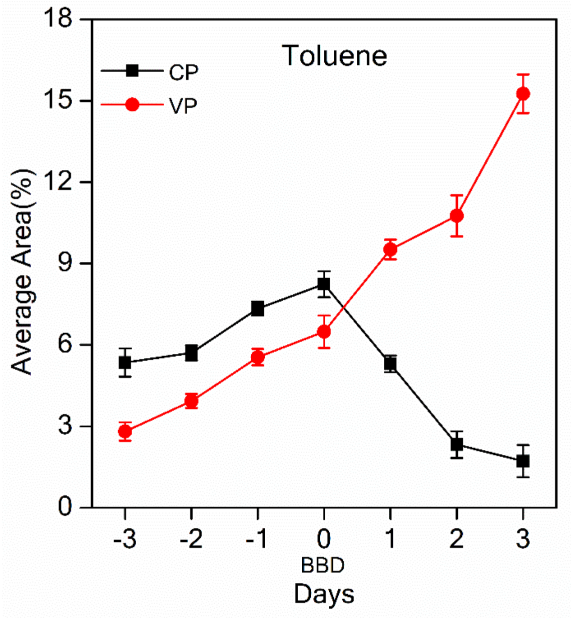

Figure S10. The average area, expressed as a percentage of all analytes, of toluene; $n = 3$, ±S. E.

Table S5. Volatile compounds from this study which are identified only by molecular features (hydrocarbon or alcohol-containing) along with peaks observed in the GC experimental results which were unable to be identified.

Tentative Compounds	Types	Retention Time (min)	Packaging	Characteristic *m/z* (fragment)	M.A. (ppm)	Calculated I	ΔI	Ref.
2,5-norbornadiene (C_7H_8)	Alkene	7.41	CP	92.0608	13.6	804	4	[19]
2,6-dimethyloctane ($C_{10}H_{22}$)	Saturated Alkane	8.08	VP	113.1299 (C_8H_{17})	2.78	869	11	[21]
3,7-dimethyl-1-octene ($C_{10}H_{20}$)	Unsaturated Alkene	8.15	MAP	97.0644 (C_6H_{13})	11.34	873	4	[18]
Cyclohexanol ($C_6H_{12}O$)	Alcohol	8.67	CP	82.0771 (C_6H_{10})	7.33	911	3	[20]
Unidentified		9.92	MAP					
Unidentified		10.08	VP					
Unidentified		10.46	VP					
Unidentified		12.29	VP					
2,4-dimethyldecane ($C_{12}H_{26}$)	Saturated Alkane	12.88	CP	112.1239 (C_8H_{16})	6.71	1091	5	Present work
2,2-trimethyldecane[c] ($C_{12}H_{26}$)	Alkane	13.64	CP	112.1265 (C_8H_{16})	3.17	1131	1	Present Work
Unidentified		22.82	CP					

Shown in the bracket is the characteristic m/z, which was used for identification if the molecular ion was not detected. [b]ΔI = |Experimental I – NIST Reference I|. The same stationary phase reference I value was used, but equivalent conditions cannot be guaranteed. [c]Compounds were identified from their fragment ions and the formula of corresponding fragment ions are given. [d] Analytes have been previously reported. The fragment m/z ion, I and ΔI values, and comparison to NIST library database are supportive of the isomers noted.

REFERENCES

1. Galgano, F.; Favati, F.; Bonadio, M.; Lorusso, V.; Romano, P. Role of Biogenic Amines as Index of Freshness in Beef Meat Packed with Different Biopolymeric Materials. *Food Res. Int.* **2009**, *42* (8), 1147–52
2. Karpas, Z.; Tilman, B.; Gdalevsky, R.; Lorber, A. Determination of Volatile Biogenic Amines in Muscle Food Products by Ion Mobility Spectrometry. *Anal. Chim. Acta* **2002**, *463* (2), 155–163.
3. Kaniou, I.; Samouris, G.; Mouratidou, T.; Eleftheriadou, A.; Zantopoulos, N. Determination of Biogenic Amines in Fresh Unpacked and Vacuum-Packed Beef during Storage at 4°C. *Food Chem.* **2001**, *74* (4), 515–519.
4. Ruiz-Capillas, C.; Jimènez-Colmenero, F. Biogenic Amines in Meat and Meat Products. *Crit. Rev. Food Sci. Nutr.* **2005**. 44(7-8):489–599.
5. Flores, M.; Olivares, A.; Dryahina, K.; Španˇl, P. Real Time Detection of Aroma Compounds in Meat and Meat Products by SIFT-MS and Comparison to Conventional Techniques (SPME-GC-MS). *Curr. Anal. Chem.* **2013**, *9* (4), 622–630.
6. Kosowska, M., A. Majcher, M.,Fortuna, T., Majcher, M. A., Fortuna, T. Volatile Compounds in Meat and Meat Products. *Food Sci. Technol.* **2017**, *37* (1), 1–7.
7. Mayr, D.; Margesin, R.; Klingsbichel, E.; Hartungen, E.; Jenewein, D.; Schinner, F.; Märk, T. D. Rapid Detection of Meat Spoilage by Measuring Volatile Organic Compounds by Using Proton Transfer Reaction Mass Spectrometry. *Appl. Environ. Microbiol.* **2003**, *69* (8), 4697–4705.
8. Bueno M, Resconi VC, Campo MM, Ferreira V, Escudero A. Development of a robust HS-SPME-GC-MS method for the analysis of solid food samples. Analysis of volatile compounds in fresh raw beef of differing lipid oxidation degrees. *Food Chem*. **2019**, 281, 49–56.
9. Du, M.; Hur, S. J.; Nam, K. C.; Ismail, H.; Ahn, D. U. Volatiles, Color, and Lipid Oxidation of Broiler Breast Fillets Irradiated before and after Cooking. *Poult. Sci.* **2001**, *80* (12), 1748–1753.
10. Zareian, M.; Böhner, N.; Loos, H. M.; Silcock, P.; Bremer, P.; Beauchamp, J. Evaluation of Volatile Organic Compound Release in Modified Atmosphere-Packaged Minced Raw Pork in Relation to Shelf-Life. *Food Packag. Shelf Life* **2018**, 18, 51–61.
11. Resconi, V. C.; Bueno, M.; Escudero, A.; Magalhaes, D.; Ferreira, V.; Campo, M. M. Ageing and Retail Display Time in Raw Beef Odour According to the Degree of Lipid Oxidation. *Food Chem.* **2018**, *242*, 288–300.
12. Tao, N. P.; Wu, R.; Zhou, P. G.; Gu, S. Q.; Wu, W. Characterization of Odor-Active Compounds in Cooked Meat of Farmed Obscure Puffer (Takifugu Obscurus) Using Gas Chromatography-Mass Spectrometry-Olfactometry. *J. Food Drug Anal.* **2014**, *22* (4), 431–438.
13. Van Ba Hoa PN, Cho SH, Kang SM, Kim YS, Moon SS, Choi YM, Kim JH, Seol KH. Quality characteristics and flavor compounds of pork meat as a function of carcass quality grade. *Asian-australas. J. Anim. Sci*. **2019**, 32(9):1448.
14. Insausti, K.; Beriain, M. J.; Gorraiz, C.; Purroy, A. Volatile Compounds of Raw Beef from 5 Local Spanish Cattle Breeds Stored under Modified Atmosphere. *J. Food Sci.* **2002**, *67* (4), 1580–1589.
15. Legako, J. F.; Cramer, T.; Yardley, K.; Murphy, T. J.; Gardner, T.; Chail, A.; Pitcher, L. R.; Macadam, J. W. Retail Stability of Three Beef Muscles from Grass-, Legume-, and Feedlot-Finished Cattle. *J. Anim. Sci.* **2018**, *96* (6), 2238–2248.
16. Wang, X.; Zhu, L.; Han, Y.; Xu, L.; Jin, J.; Cai, Y.; Wang, H. Analysis of Volatile Compounds between Raw and

Cooked Beef by HS-SPME–GC–MS. *J. Food Process. Preserv.* **2018**, *42* (2), 1–8.

17. Karabagias, I. K. Volatile Profile of Raw Lamb Meat Stored at 4 ± 1 °C :The Potential of Specific Aldehyde Ratios as Indicators of Lamb Meat Quality.*Foods* **2018**,7(3), 40.
18. Harrington BA, inventor; ExxonMobil Chemical Patents Inc, assignee. Process for producing elastic thermoplastic α-olefin/cyclic olefin copolymers. United States patent US 5,837,787. **1998**
19. Ahn DU, Jo C, Olson DG. Analysis of volatile components and sensory characteristics of irradiated raw pork. *Meat Sci*, **1999**, *54*, 209–215.
20. Park, S. Y.; Yoon, Y. M.; Schilling, M. W.; Chin, K. B. Evaluation of Volatile Compounds Isolated from Pork Loin (Longissimus Dorsi) as Affected by Fiber Type of Solid-Phase Microextraction (SPME), Preheating and Storage Time. *Korean J Food Sci Anim Resour* **2009**, *29* (5), 579–589.
21. Laird, H. L. Millenial's Perception of Beef Flavor, **2015**.(Doctotal Dissertation),Texas A&M.
